# 

**Published:** 1871-04

**Authors:** 


### THE AMERICAN MEDICAL ASSOCIATION.

We have already called attention to the meeting of the National Association, commencing on the 2d of May, at 11 o'clock, at San Francisco, California. 1871

This Association is justly regarded the great Congress of the profession, having representatives from every State and Territory of the Union. The great ends sought to be accomplished by such an organization, are to unite the profession upon just principles for its government, and to advance the knowledge and cause of science. There remain many things still for the Association to do, in order to place its power and influence beyond question or controversy. It should announce its principles without reference to localities, sections or individual interests—principles for the good of the entire profession as a body. Individual rights, as well as obligations to the laws of the profession, should be clearly defined upon all doubtful points. It should, in our opinion, announce its position upon medical education by a positive enactment, which should in future govern all medical institutions represented in it. Having asserted the right to control medical education, it should clearly define its requirements of all institutions claiming to be regular.

We trust that in all its deliberations, wisdom and moderation, founded upon justice and truth, will mark its proceedings. Principle should not be sacrificed at the behest of expediency, or truth shorn of its power by being ignored. We hope ever to bear witness to its loyalty to these cardinal principles—to feel that it still venerates the laws of its own creation, and that it will assert its authority and power to shield the interests and guard the honor of a profession it so proudly represents.

The permanent Secretary of the Association, Dr. Wm. B. Atkinson, requests the Secretaries of all medical organizations to forward lists of their delegates as soon as elected. Institutions desiring to be represented are required to transmit to the Secretary, with the credentials of its delegates, evidence of their existence, capacity and good standing.

### THE MACON MEDICAL ASSOCIATION.

The proceedings of this Society have been forwarded us for publication, which, we are sorry to say, reached us too late to be placed in this number of the Companion. It will appear in our next. We hope our friends of Macon will send us, each month, extracts of their minutes, with report of cases, &c.

---

### THE FULTON COUNTY MEDICAL SOCIETY.

The proceedings of this Society have been unavoidably left out of this issue. Our friends must bear with us.

### OUR EXCHANGES.

*Richmond and Louisville Medical Journal:* Published at Louisville, Ky., by E. S. Gaillard, M. D., editor and proprietor, Professor of Principles and Practice of Medicine in the Louisville Medical College. This is the largest monthly in America, and one of the best, its pages showing great excellence, both in arrangement and matter. The editorial department is unusually ably conducted, and the Journal placed by its gifted editor upon the highest summit of professional law. This journal, so scientific in its scope, and so elevated in tone, should have a circulation throughout the length and breadth of the country. It is an earnest, faithful advocate of truth and law, and should be in the hands of every physician. Terms: $5.00 in advance.

*Buffalo Medical and Surgical Journal:* Published by Julius F. Miner, M. D., editor and proprietor, Professor of Special Surgery in the Buffalo Medical College. The editor is an accomplished gentleman and scholar, and his journal sustains his character as such. Terms: $3.00 in advance.

*The Baltimore Medical Journal and Bulletin:* E. Lloyd Howard, M. D.; T. S. Latimer, M. D., editors and proprietors. Issued monthly. Terms: $4.00 a year, in advance. This is a neat and well printed journal. The number before us is excellent, being filled with original and selected articles of very great value to the professional reader.

*The Cincinnati Medical Refertory:* Published by the Medical Journal Association, D. D. Bramble, M. D., Agent. Edited by J. A. Thacker, M. D. This is a sprightly journal, full of vim and vigor, and up to the times in medical literature.

*The Medical Gazette and Weekly Review of the Medical Sciences:* Published every Saturday at 109 Nassau street, New York, by Henry W. Turner. $3.50 per annum. This is a very valuable weekly. It should have a large circulation.

*The American Chemist*, a monthly Journal of Theoretical, Analytical and Technical Chemistry: Published by Wm. Baldwin & Co., 177 Broadway, New York, and edited by Charles F. Chandler, Ph. D., and W. H. Chandler. This journal contains much scientific matter, useful to the physician, and of great practical importance to druggists. Terms: $5.00 per annum in advance.

*The Journal of Materia Medica*, devoted to Materia Medica, Pharmacy and Chemistry: Conducted by Joseph Bates, M. D., and H. A. Tilden. Lebanon, New York. This is a neat journal of thirty-two pages, and extremely useful, both to physicians and druggists. It is the representative of the pharmaceutical preparations of that excellent and reliable manufacturing establishment, Tilden & Co. Much could be said as to the purity and reliability of the drugs prepared by this firm. Terms of the journal: $1.00 per annum.

*The Phgsician and Pharmaceutist:* Published quarterly, by Reed, Carnrick & Andrus, No. 122 Liberty street, New York. Edited by G. Treskotis, M. D. This is an excellent quarterly. We regret it is not a monthly instead. The establishment publishing it is one of the most extensive and worthy drug firms of the Metropolis.

*The Boston Journal of Chemistry:* Published by J. R. Nicholds, M. D., 150 Congress street, Boston, Mass. This is a valuable monthly, edited by a highly educated gentleman and chemist. Its pages are filled with useful articles, literary, scientific and medical. The excellent firm of J. R. Nicholds & Co. should be better known South. Terms of journal: $1.00 per annum.

*The Herald of Health and Journal of Physical Culture:* Published by Wood & Holbrook, 13 and 15 Laight street, New York. This journal contains a large amount of useful and practical information to the physician—much that he would appreciate and apply in the interests of humanity. We thank the publishers for their kindness in forwarding us a complete set of their journal for 1870. Terms: $2.00 per annum.

*Good Health*, a Journal of Physical and Mental Culture: Published by Alexander Moore, 11 Broomfield street, Boston. This is also a journal which the physician will read with profit. We thank the publishers for copies for the year 1870. Terms: $2.00 per annum.

*The Detroit Review of Medicine and Pharmacy:* George P. Andrews, M. D., Editor and Proprietor; W. H. Lathrop, M. D. Associate Editor. This is an excellent journal, edited with remarkable care and ability, and filled with valuable matter. Published in Detroit, Mich., at $2,00 per annum.

*The Pharmacist and Chemical Record:* A Monthly Journal, devoted to Pharmacy, Chemistry, and the Collateral Sciences. Published by the Chicago College of Pharmacy. Edited by N. Gray Bartlett and Albert E. Ebert. This journal is of great value to the scientific physician desirous of keeping up with the rapid progress of Chemistry and Pharmacy. It should be in the hands of every druggist.

We have received the Chicago Medical Times, a monthly journal, devoted to the interests of Eclectic Medicine and Surgery. Edited by John E. Hurlbut, M. D., which we place upon our exchange list. Terms of journal: $2.00 per annum.

We have also received the *Eclectic Medical Journal*, edited by John M. Scudder, M. D., Cincinnati, Ohio. This Journal represents the interests and objects of the eclectic practice—however misapplied the term—with much ability and great industry. Terms $2.00 per annum.

The American Medical Eclectic Review is placed upon our exchange list. It is published in New York. Edited by Rob't. S. Newton, M. D., P. Albert Morrow, M. D., and Alexander Wilder, M. D. Like the above, this Journal is devoted to Eclecticism. Terms, $2.00.

*The Semi-Weekly Medical and Surgical Reporter:* Published at Griffin, Ga., and edited by E. F. Knott, M. D., and J. J. Knott, M. D. We received the first number of this journal, and had a notice of it in type, but receiving no others, and having heard that it had been suspended, we did not notice it. We are pleased to hear, however, that it still lives, and is growing in public favor. We wish the enterprise much success. Our friends should not overlook the COMPANION by not sending their journal.

